# Densities in the left innominate vein after removal of an implantable venous device: a case report

**DOI:** 10.1186/1752-1947-6-180

**Published:** 2012-07-02

**Authors:** James van Bastelaar, Caroline H C Janssen, Eveline de Bont, Nicole M Blijlevens, Robertine van Baren

**Affiliations:** 1 Department of Paediatric Surgery, University Medical Center Groningen, PO Box 30.001, 9700, RB, Groningen, The Netherlands; 2 Department of Radiology, University Medical Center Groningen, PO Box 30.001, 9700, RB, Groningen, The Netherlands; 3 Department of Paediatric Oncology, University Medical Center Groningen, PO Box 30.001, 9700, RB, Groningen, The Netherlands; 4 Department of Haematology, University Medical Center Nijmegen, 6500, HB, Nijmegen, The Netherlands

## Abstract

**Introduction:**

Pericatheter calcifications are unusual and rare after removal of indwelling central venous catheters with few reports in the literature. We present a case of a woman with calcifications in her left innominate vein after removal of an implantable venous device.

**Case presentation:**

A venous access port was surgically placed for intravenous chemotherapy in a 19-year-old Caucasian woman who had been diagnosed with acute lymphoblastic leukemia. She developed a fever three and a half years after placement, and the venous access port was removed as it was seen as the only focus for her fever. In the year following its removal, a computed tomography scan was ordered due to a clinical suspicion of deep venous thrombosis of her left arm. The computed tomography scan revealed a hyperdense structure in the left innominate vein with thrombosis. It was concluded that this was a foreign body, a retained catheter fragment after removal of the catheter. After three-dimensional reconstructions were performed, it was determined that these hyperdense structures were calcifications in the left innominate vein that resembled a foreign body.

**Conclusions:**

Differentiating between intravenous thrombotic calcification and a retained catheter tip after removal can be challenging, even with modern day diagnostic tools. Care should be taken to document the length of the catheter upon placement and upon removal. In this manner, unnecessary surgical exploration can be avoided. We would like to highlight the importance of these diagnostic considerations for radiologists and oncologists.

## Introduction

Indwelling central venous catheters are used regularly in children with hematological malignancies. Intravenous calcifications after removal of indwelling central venous catheters or implantable venous devices are rare, with few reports in the literature [1,2]. The use of conventional computed tomography (CT) does not necessarily make it easier to differentiate between the presence of a foreign body (tip of a catheter) or intravascular calcifications.

We present a case of a woman with calcifications in her left innominate vein after removal of an implantable venous device. This finding was initially mistaken for a foreign body (incomplete removal of the indwelling central venous catheter).

## Case presentation

A 19-year-old female Caucasian patient was diagnosed with acute lymphoblastic leukemia at the age of 15. Treatment was initiated according to the acute lymphoblastic leukemia (ALL)-10 protocol. A venous access port for intravenous infusion of chemotherapy was surgically placed with the tip of the catheter in her superior vena cava via her left subclavian vein. Treatment was continued for two years.

Ten months after her final treatment with chemotherapy, our patient relapsed and induction chemotherapy was administered according to the ALL-R3 protocol. This was complicated by renal failure and encephalopathy and therefore mechanical ventilation and dialysis were initiated. After clinical recovery, her treatment was switched to an alternative re-induction regimen.

During follow up treatment with 6-mercaptopurine, methotrexate, cytarabine, idarubicin, etoposide and cyclophosphamide, our patient presented to the outpatient clinic with a fever and general malaise. Blood work revealed a C-reactive protein level of 462 mg/L. The differential diagnosis included sinusitis, deep neck infection and line infection. Blood cultures were negative. A CT scan of her neck was ordered, which revealed a submental abscess extending into the submandibular space. Her skin was incised and the underlying abscess was drained.

Our patient’s fever persisted, and as the central venous catheter was still *in situ* it was seen as the only possible remaining cause of infection. The catheter was surgically removed without any problems, three and a half years after placement. Cultures of the catheter tip were negative, however her fever persisted and she still showed signs of ongoing infection. This inflammatory response, possibly caused by immune reconstitution, was treated with colchicine and Indomethacin. Her fever subsided and the colchicine and Indomethacin were subsequently stopped, even though her C-reactive protein level remained high (169 mg/L). Three months after removal of the indwelling central venous catheter, she underwent a stem cell transplantation.

**Figure 1:**
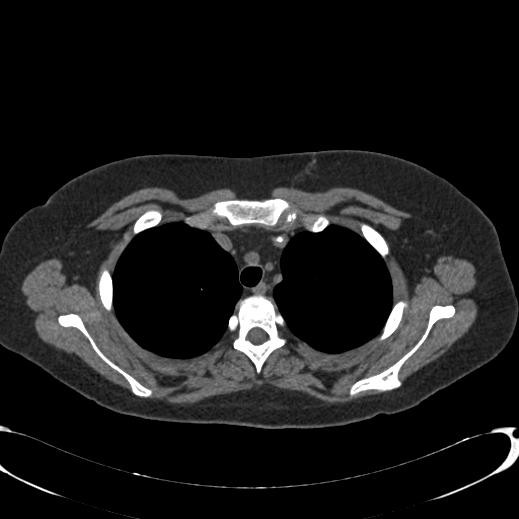
**Transverse plane computed tomography image of the chest.** An area of visible density in the left innominate vein as depicted on the transverse plane

**Figure 2:**
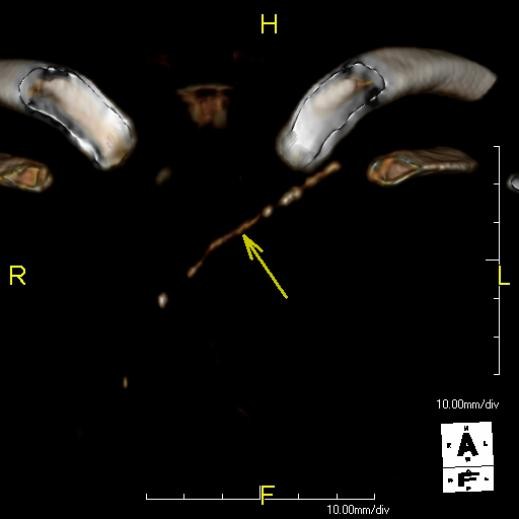
**Three-dimensional reconstruction.** A hyperdense line of variable density is depicted with an irregular surface and interrupted continuity in the left innominate vein

During stem cell transplantation, clinical suspicion arose for deep venous thrombosis of her left arm. A CT scan revealed a hyperdense structure in her left innominate vein, with thrombosis (Figure 1). After a conferral with the radiologist and clinicians, it was concluded that this hyperdense lesion was a foreign body, most likely the tip of the central venous catheter which had previously been (incompletely) removed. However, on conventional chest radiographs no foreign body was visible. Due to this inconsistency, three-dimensional reconstructions were done; it became clear that this ‘hyperdense line’ was of variable density, had an irregular surface and the continuity was interrupted at certain points (Figure 2). It was now concluded that these hyperdense structures were calcifications that had developed after line removal in an old (infected) thrombus in her left innominate vein, which resembled a foreign body. She was treated with low molecular weight heparin and antibiotics, after which her fever subsided.

## Discussion

Complications after the insertion of central venous catheters are well documented and generally include pneumothorax, hemothorax, hematoma, thrombosis and infection [3]. There are few reports concerning complications that occur after removal of central lines. Jones and Giacomantonio describe a retrospective study of 136 central line removals. There were three complications of retained catheter fragments, possibly due to fibrin sheath formation and pericatheter calcification [4]. The formation of a central venous catheter thrombus is likely multifactorial: vessel injury upon placement; venous stasis caused by the presence of the central venous catheter; cancer-related hypercoagulability; and prolonged placement [1]. Formation of calcifications seems more likely in patients receiving total parenteral nutrition and those with long-term indwelling catheters. Thrombotic pericatheter calcifications can, however, occur in patients with central lines being used solely for the administration of chemotherapy [5].

In this case, it is retrospectively postulated that an infected thrombus must have been present in the left innominate vein upon removal of the venous access port. After removal, calcifications formed where the catheter had previously been positioned. Prolonged treatment with antibiotics eventually caused the infection in the thrombus to subside. The placement of a new central venous line for stem cell transplantation via the left subclavian vein caused fresh thromboses to form on the old thrombus. Therefore, diagnostic CT was required. Initial clinical and radiographical assessments were misleading, suggesting incomplete removal of the central venous catheter. A previous infected thrombus with calcifications can mimic the presence of venous foreign bodies.

## Conclusion

Differentiating between intravenous thrombotic calcification and a retained catheter tip after removal can be challenging, even with modern day diagnostic tools. It is important to document the length of the catheter upon placement and removal. All indwelling catheters should be carefully inspected upon removal. This allows the differential diagnosis to be narrowed down and unnecessary surgical exploration can be avoided. There are several publications focusing on this phenomenon; however, the presence of pericatheter calcifications is not always considered by radiologists and oncologists. We would like to highlight the importance of these diagnostic considerations in this clinical setting.

## Consent

Written informed consent was obtained from the patient for publication of this case report and accompanying images. A copy of the written consent is available for review by the Editor-in-Chief of this journal.

## Competing interests

The authors declare that they have no competing interests.

## Authors’ contributions

JvB retrieved data, did background research and wrote the article. CHCJ retrieved radiographic data, did background research and edited the manuscript. EdB interpreted patient data regarding hematological disease and edited the manuscript. NMB retrieved patient data and edited the manuscript. RvB retrieved data, and edited and supervised the manuscript. All authors read and approved the final manuscript.
